# 1614. Efficacy and Safety of Adjusted Renal Dosing of Tenofovir DF Versus Abacavir Switching in HIV Patients with Decreased Glomerular Filtration Rate After Receiving Tenofovir DF-Based Regimen

**DOI:** 10.1093/ofid/ofad500.1449

**Published:** 2023-11-27

**Authors:** Eakkawit Yamasmith, Rujipas Sirijatuphat, Winai Ratanasuwan, Peerawong Werarak

**Affiliations:** Police General Hospital, Bangkok, Krung Thep, Thailand; Faculty of Medicine Siriraj Hospital, Mahidol University, Bangkok, Thailand, Bangkok, Krung Thep, Thailand; Faculty of Medicine, Siriraj Hospital, Mahidol University, Bangkok, Krung Thep, Thailand; Faculty of Medicine Siriraj Hospital, Mahidol University, Bangkok, Thailand, Bangkok, Krung Thep, Thailand

## Abstract

**Background:**

A switch from tenofovir disoproxil fumarate (TDF) to abacavir (ABC)-based therapy is one of treatment options for HIV-infected patients with decreased glomerular filtration rate (eGFR) after receiving a TDF-based regimen. However, ABC may not be available in some hospitals in Thailand. TDF dose reduction showed a significant improvement of eGFR without virological failure. This study aimed to compare the efficacy and safety of TDF dose reduction versus ABC switching for the treatment of patients with decreased eGFR after receiving TDF.

**Methods:**

This open-label, randomized, non-inferiority study was conducted at Siriraj Hospital, Thailand, during March 2019 to May 2021. Eligible participants were HIV-suppressed patients aged ≤18 years with decreased eGFR (eGFR 30-60 mL/min by Cockcroft-Gault equation) after receiving TDF-based therapy. Patients were randomized (1:1 by block of four) to a TDF dose-adjusted regimen or an ABC-based regimen. The primary outcome of study was the change in eGFR at 24 weeks after regimen modification. The non-inferiority margin was -12 mL/min. Other outcomes were the virological efficacy and safety at 24 weeks after regimen modification.

**Results:**

30 patients were enrolled (15 patients in the TDF dose-adjusted regimen group and 15 patients in the ABC-based regimen group). The distribution of age, sex, CD4 count, serum creatinine, eGFR and duration of TDF treatment were similar between both groups. The mean change of eGFR (SD) at 24 weeks after regimen modification were +1.87 (7.90) mL/min in the TDF dose-adjusted regimen group and +15.64 (6.53) mL/min in the ABC-based regimen group (P< 0.001). The difference of mean change of eGFR between both groups at 24 weeks was -13.77 mL/min (95% CI; -20.18 to -7.36 mL/min) (P< 0.001). The sustained viral suppression was observed in both groups. Two patients in the TDF dose-adjusted regimen group developed progressive kidney impairment and one of them developed Fanconi syndrome.

Baseline Characteristics

**Figure:**
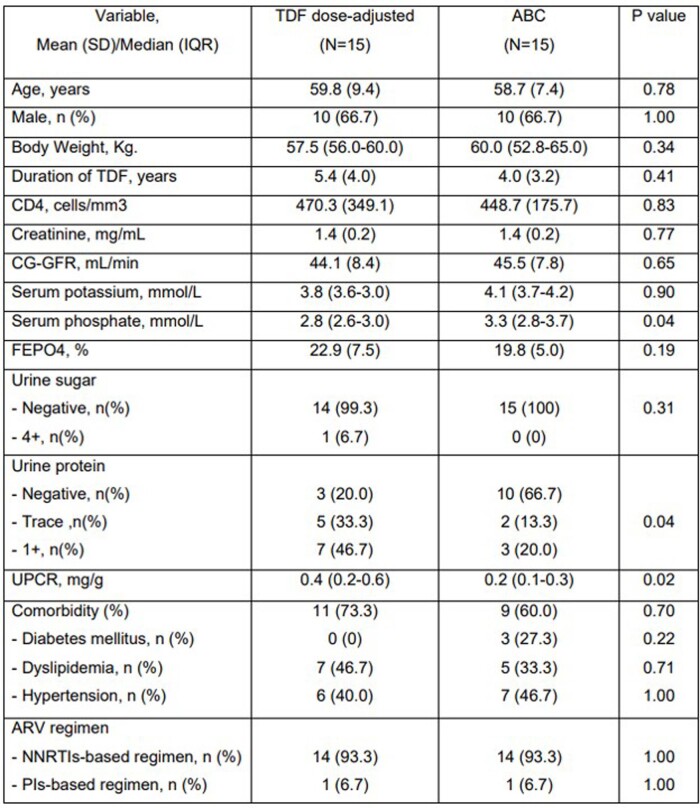

Outcomes

**Figure:**
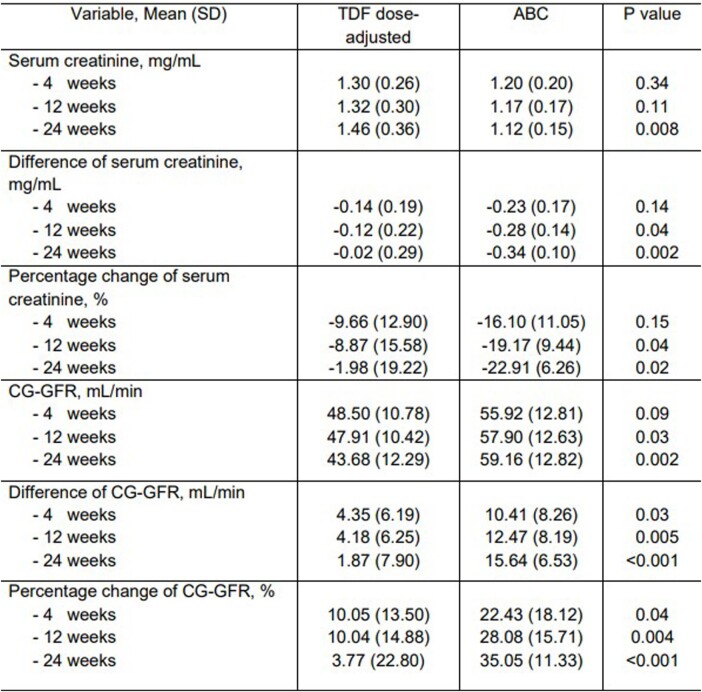

**Conclusion:**

The TDF dose-adjusted regimen demonstrated the efficacy in improving eGFR but this regimen did not prove non-inferiority over the ABC-based regimen. The progression of kidney dysfunction should be cautioned in patients who received the adjusted renal dosing of TDF.

**Disclosures:**

**Eakkawit Yamasmith**, Police General Hospital: Advisor/Consultant

